# Usefulness of Random Forest Algorithm in Predicting Severe Acute Pancreatitis

**DOI:** 10.3389/fcimb.2022.893294

**Published:** 2022-06-10

**Authors:** Wandong Hong, Yajing Lu, Xiaoying Zhou, Shengchun Jin, Jingyi Pan, Qingyi Lin, Shaopeng Yang, Zarrin Basharat, Maddalena Zippi, Hemant Goyal

**Affiliations:** ^1^Department of Gastroenterology and Hepatology, The First Affiliated Hospital of Wenzhou Medical University, Wenzhou, China; ^2^School of the First Clinical Medical Sciences, Wenzhou Medical University, Wenzhou, China; ^3^Jamil-ur-Rahman Center for Genome Research, Dr. Panjwani Centre for Molecular Medicine and Drug Research, International Center for Chemical and Biological Sciences, University of Karachi, Karachi, Pakistan; ^4^Unit of Gastroenterology and Digestive Endoscopy, Sandro Pertini Hospital, Rome, Italy; ^5^Department of Medicine, The Wright Center for Graduate Medical Education, Scranton, PA, United States

**Keywords:** random forest, nomogram, acute pancreatitis, predictor, artificial intelligence, LIME plot

## Abstract

**Background and Aims:**

This study aimed to develop an interpretable random forest model for predicting severe acute pancreatitis (SAP).

**Methods:**

Clinical and laboratory data of 648 patients with acute pancreatitis were retrospectively reviewed and randomly assigned to the training set and test set in a 3:1 ratio. Univariate analysis was used to select candidate predictors for the SAP. Random forest (RF) and logistic regression (LR) models were developed on the training sample. The prediction models were then applied to the test sample. The performance of the risk models was measured by calculating the area under the receiver operating characteristic (ROC) curves (AUC) and area under precision recall curve. We provide visualized interpretation by using local interpretable model-agnostic explanations (LIME).

**Results:**

The LR model was developed to predict SAP as the following function: -1.10-0.13×albumin (g/L) + 0.016 × serum creatinine (μmol/L) + 0.14 × glucose (mmol/L) + 1.63 × pleural effusion (0/1)(No/Yes). The coefficients of this formula were utilized to build a nomogram. The RF model consists of 16 variables identified by univariate analysis. It was developed and validated by a tenfold cross-validation on the training sample. Variables importance analysis suggested that blood urea nitrogen, serum creatinine, albumin, high-density lipoprotein cholesterol, low-density lipoprotein cholesterol, calcium, and glucose were the most important seven predictors of SAP. The AUCs of RF model in tenfold cross-validation of the training set and the test set was 0.89 and 0.96, respectively. Both the area under precision recall curve and the diagnostic accuracy of the RF model were higher than that of both the LR model and the BISAP score. LIME plots were used to explain individualized prediction of the RF model.

**Conclusions:**

An interpretable RF model exhibited the highest discriminatory performance in predicting SAP. Interpretation with LIME plots could be useful for individualized prediction in a clinical setting. A nomogram consisting of albumin, serum creatinine, glucose, and pleural effusion was useful for prediction of SAP.

## Highlights

1. An interpretable random forest model exhibited the highest discriminatory performance in SAP prediction.
2. Interpretation with LIME plots could be useful for individualized prediction in a clinical setting.
3. A nomogram comprising albumin, serum creatinine, glucose, and pleural effusion is a useful predictor of SAP.

## Introduction

Acute pancreatitis (AP) is one of the most common gastrointestinal problems for hospital admission globally (Hong et al., 2020). While most patients with AP will recover within a week of a mild course and are often self-limiting, 20% of patients progress to severe disease with a historical mortality risk as high as 30% (Trikudanathan et al., 2019). In the absence of specific treatment in the early phase, initial management of severe acute pancreatitis (SAP) focuses on supportive care such as fluid resuscitation, pain control, and nutritional support, aimed to minimize the impact of systemic inflammatory response syndrome (Lee and Papachristou, 2019). Patients with SAP often need to be transferred to the intensive care unit once organ failure occurs. Therefore, it is important to recognize predictors for severe disease in the early phase of AP, to select those patients who would benefit most from enhanced surveillance or early interventions. Early case identification and classification of disease severity could improve the clinical outcomes (Hong et al., 2021).

Many clinical scoring systems have been developed for the prediction of disease severity, such as the Ranson, chronic health evaluation (APACHE-II) score, Pancreatitis Outcome Prediction (POP) Score (Harrison et al., 2007), and Bedside Index Of Severity In Acute Pancreatitis (BISAP) (Wu et al., 2008). However, the existing scoring systems have moderate accuracy in predicting the severity of AP (Mounzer et al., 2012). Recently, Langmead et al. reported that a 5-cytokine panel consisting of angiopoietin-2, hepatocyte growth factor, interleukin-8, resistin, and soluble tumor necrosis factor receptor 1A accurately predicts persistent organ failure early in the disease process and significantly outperforms the prognostic accuracy of existing laboratory tests and clinical scores (Langmead et al., 2021). However, the test of cytokine is not routinely available, resulting in limited use in clinical practice. Several laboratory indexes such as total cholesterol (Hong et al., 2020), low-density lipoprotein cholesterol (Hong et al., 2018), albumin (Hong et al., 2017a), and blood urea nitrogen (BUN) (Lin et al., 2017) have been proposed as single predictors of severity of AP. Recently, Takeda et al. reported that fluid sequestration is a useful parameter in the early identification of SAP (Takeda et al., 2019). Yan et al. described that pleural effusion volume could be a reliable radiologic biomarker in the prediction of severity and clinical outcomes of AP (Yan et al., 2021). Gibor et al. reported that circulating cell-free DNA in patients with acute biliary pancreatitis is associated with disease markers and prolonged hospitalization time (Gibor et al., 2020). However, these single prediction markers are easy to use in practice but lack high accuracy.

Recently, artificial intelligence methods are also being used in the clinical setting for disease prediction or aiding in making decisions. Among several methods, a random forest (RF) is a group of many decision trees, each of which is characterized by a tree-like structure (Genuer and Poggi, 2020). It will randomly choose features and make observations, build a forest of decision trees, and then average out the results (James et al., 2013). RF allows considering qualitative and quantitative explanatory variables together, without preprocessing (Genuer and Poggi, 2020). Random forests are adapted to both supervised classification problems and regression problems (Genuer and Poggi, 2020). In addition, RF can handle datasets with many predictor variables, while also performing very well. Additionally, it can obtain variable importance ranking when used for prediction modeling (Speiser et al., 2019). RF, as a traditional machine learning method, has been shown to outperform other techniques for sets of features in a variety of different settings. RF has recently demonstrated high performance in risk classification and disease prediction (Yu et al., 2021). Lo et al. developed RF model for forecasting allergenic pollen in North America (Lo et al., 2021). Lin et al. reported that using the RF model could predict environmental risk factors in relation to health outcomes among school children from Romania (Lin et al., 2021b). Roguet et al. reported that RF classification with 16S rRNA gene amplicons offers an accurate solution for identifying host microbial signatures (useful in detecting human and animal fecal contamination in environmental samples) (Roguet et al., 2018). Yang et al. provided an RF prediction model for 3-year risk assessment of cardiovascular disease (Yang et al., 2020).

However, to the best of our knowledge, the use of RF model in predicting disease severity in patients with AP has not been performed yet. The aim of this study was to develop an RF model and compare it with a traditional logistic regression (LR) model for prediction of SAP.

## Patients and Methods

### Inclusion and Exclusion Criteria

We conducted a *post-hoc* analysis of a previously reported retrospective cohort study in the First Affiliated Hospital of Wenzhou Medical University in mainland China (Hong et al., 2018). Patients with AP admitted to the First Affiliated Hospital of Wenzhou Medical University within 72 h of symptom onset (from April 1, 2012 to December 31, 2015) were consecutively enrolled in the study (Hong et al., 2020). The diagnosis of AP was based on the presence of two of the following three features: (1) abdominal pain consistent with AP; (2) serum amylase and/or lipase more than three times that of the normal; (3) abdominal imaging findings (Hong et al., 2020). As previously described (Hong et al., 2020), exclusion criteria included endoscopic or trauma related pancreatitis, chronic pancreatitis, pancreatic tumor, history of surgery operation/taking hypolipidemia drugs, malnutrition, and chronic liver or renal disease.

### Data Collection

The clinical and laboratory data on admission were obtained with data collection forms from electronic medical records. These data included blood chemica+l analysis, liver, and renal function testing, glucose, lipids, coagulation testing, serum calcium, C-reaction protein, and pleural effusion (Hong et al., 2020).

### Definition of Severity and Study Endpoint

SAP is defined as a persistent organ failure (>48 h) in patients. Organ failure for this study was defined according to a Marshall score ≥2, meaning that at least one organ system (respiratory, cardiovascular, renal) must be affected (Hong et al., 2018). The primary study endpoint was the occurrence of SAP during hospitalization.

### Sample Size and Missing Values

The calculation of the sample size of this study was according to our previous study (Hong et al., 2020). There were missing values in serum calcium and C-reactive protein data. To handle this issue, missing values were imputed using Multiple Imputations by Chained Equations (MICE) when performing LR and RF analysis (Royston, 2005). The MICE has emerged as one of the principal statistical approaches for dealing with missing data. The missing values were replaced by the estimated plausible values to create a “complete” dataset (Royston, 2005).

### Statistical Analysis

Categorical values were described by count and proportions and compared by the χ2 test or Fisher’s exact test. According to the results of the Shapiro-Wilk test, continuous values were expressed by mean ± SD or median and Inter Quartile Range (IQR) and compared using Student’s t-test or the Wilcoxon’s non-parametric test. The discriminative power of the predictor was assessed by calculating the area under the receiver operating characteristic (ROC) curves (AUC) (Hong et al., 2019). A variable with an AUC above 0.7 was considered useful (Hong et al., 2017b).

The data samples (of 648 patients) were randomly split into training and test sets according to a division of 3:1 (487 *vs*. 161 patients). The RF model was developed on the training set and independently validated on the test set by using “randomForest” (Liaw and Wiener, 2002) and “caret” package (Kuhn, 2008). When we built and tuned the RF model on a training set, we used tenfold cross-validation as the resampling method to avoid overfitting of the model in new data (Kuhn, 2008). The training set was divided into 10 equal-sized sub-samples in which 9 sub-samples were for the training and the remaining ones for testing over all possible permutations. Analysis was repeated 10 times (folds) (Hong et al., 2019). The AUC was calculated for each of the 10 analyses, using only the respective test data (Hong et al., 2019). Then this iteration process was repeated 10 times. At last, the mean AUC with 95%CI, as well as area under precision recall curve was calculated and compared (Saito and Rehmsmeier, 2015; Hong et al., 2019).

After training the RF model, a general approach of interpretability is to identify important variables (features) in the model (Staniak and Biecek, 2018). The RF algorithm estimates the importance of a variable by looking at how much prediction error increases when Out-Of-Bag (OOB) data for that variable are permuted, while all others are left unchanged (Liaw and Wiener, 2002; Genuer and Poggi, 2020). The variable importance is a global explanation of relative importance of each feature in the RF model (Kuhn, 2008). Variables having high importance are drivers of the outcome and their values have a significant impact on the result values.

To overcome the black box problem of the RF model output and improve its interpretability, the local interpretable model-agnostic explanations (LIME) plot was used to explain the individualized prediction (Deshmukh and Merchant, 2020). LIME is a novel explanation technique that explains the predictions of any classifier in an interpretable and faithful manner, by learning an interpretable model locally around the prediction (Ribeiro et al., 2016). The training of the local interpretable model involves giving weight to the disturbance input, followed by the observation of the general (black box) model output, which gives a basis for interpretation of the prediction results (Pan et al., 2020). This feature is deemed important if perturbations at the local level produce a change in the general model while the value of the target feature is determined by the level of change it determines (Bramhall et al., 2020). Local explanation detects variables’ contribution at the local level. In other words, LIME could provide easily understood explanations of clinical factors in the RF models, which contribute to each prediction for the individual patient (Petch et al., 2022). LIME was performed by using the “lime” package (“Lime: Local Interpretable Model-Agnostic Explanations, 2021”, http://cran.itam.mx/web/packages/lime/index.html), in which two types of inputs (tabular and text) are supported (Pedersen and Benesty, 2021).

A forward-conditional stepwise LR analysis was also applied on the training set. The conditional probabilities for stepwise entry and removal of a factor were 0.05 and 0.06, respectively (Hong et al., 2020). Based on the results of LR, a nomogram was developed to predict SAP. Model calibration, reflecting the link between predicted and observed risk, was evaluated by the Hosmer-Lomeshow goodness of fit test, as well as plotting the predicted *vs*. observed deciles of predicted risk (Hong et al., 2017b). Odds ratios (OR) were calculated, with 95%CI.

We selected the best cut-off point, where the number of true positives was the highest possible (sensitivity >90%). This was done by selecting a threshold value at a point where the longest increase in the specificity of the slope declines for all models and scores. The sensitivity, specificity, and accuracy were calculated and compared (Saito and Rehmsmeier, 2015).

A two-tailed P-value of less than 0.05 was considered statistically significant. All statistical analysis were performed in the R and STATA software. A data flow diagram of our study is shown in **Supplementary Figure S1**.

## Results

### Baseline Characteristics

Of all the patients, the hospital mortality was 1.54%. There were 247 (58.8%) men and their median age was 53 (42.0–64.5) years. The most common etiology of AP was biliary (42.4%). The median time interval between onset and admission was 2 (IQR 1-2) days. Of these patients 10% developed SAP during hospitalization. The median length of the hospital stay was 10 (IQR 7-14) days. The baseline characteristics of the patients in the training and test sets are shown in **Table 1**.

**Table 1:** Comparison of clinical and laboratory findings among patients, with and without SAP (training sample set).

Variable	Training set (n = 487)	Test set (n = 161)	P-value
Age, years (IQR)	47 (37,61)	49 (36,64)	0.501
Male sex, N (%)	301 (61.81)	103 (63.98)	0.623
Duration of symptoms (days, IQR)	1.83 ± 0.80	1.78 ± 0.78	0.515
BMI, kg/m^2^ (IQR)	23.5 (21.1-26.3)	23.9 (21.5-21.5)	0.573
SIRS, N (%)	191 (39.22)	65 (40.37)	0.795
Biliary etiology, N (%)	207 (42.51)	68 (42.24)	0.584
Laboratory findings			
Hematocrit (l/l)	0.42 (0.38-0.46)	0.42 (0.38-0.46)	0.693
Platelets (109/L)	199 (161-233)	195 (157-233)	0.472
Prothrombin time, s (IQR)	13.8 (13.1-14.6)	13.7 (13.0-14.5)	0.278
Albumin, g/L (IQR)	36.3 (32.6-39.9)	36.4 (34.0-39.8)	0.191
Total bilirubin, mmol/L (IQR)	20 (14-31)	20 (13-32)	0.916
ALT, U/L (IQR)	43 (19-119)	31 (19-82)	0.055
AST, U/L (IQR)	39 (22-88)	28 (19-71)	0.012
Glucose, mmol/L (IQR)	7.9 (6.3-10.5)	8.4 (6.7-11.3)	0.128
Serum creatinine, μmol/L (IQR)	64 (54-77)	64 (55-76)	0.882
BUN, mmol/L (IQR)	4.8 (3.7-6.1)	4.9 (4.0-6.2)	0.346
Total cholesterol, mmol/L (IQR)	4.79 (3.8-6.2)	4.8 (3.8-6.1)	0.970
HDL, mmol/L (IQR)	1.0 (0.7-1.3)	1.0 (0.8-1.3)	0.461
LDL, mmol/L (IQR)	2.5 (1.9-3.2)	2.2 (1.8-3.0)	0.100
Triglyceride (mg/dL), mmol/L (IQR)	1.3 (0.8-3.4)	1.3 (0.8-3.6)	0.995
Serum calcium, mmol/L (IQR)	2.7 (2.1-2.3)	2.2 (2.1-2.3)	0.051
C-reactive protein, mg/L (IQR)	35.0 (11.7-90.0)	29.4 (8.7-85.3)	0.415
Pleural effusion, N (%)	89 (18.28)	35 (21.74)	0.333
Patients with SAP, N (%)	49 (10.1)	16 (9.9)	0.0092
Length of hospital stay, days (IQR)	10 (7-13)	11 (7-15)	0.964
Hospital mortality, N (%)	9 (1.85)	1 (0.62)	0.274

Data were mean ± standard deviation, or numbers and percentages, or median (25th–75th percentile), as appropriate. N, number; IQR, interquartile range; BMI, body mass index; SIRS, systemic inflammatory response syndrome; ALT, alanine aminotransferase; AST, aspartate aminotransferase; HDL, high-density lipoprotein cholesterol; LDL, low-density lipoprotein cholesterol.

### Univariate Analysis on the Training Sample

As shown in **Table 2**, 16 variables, namely, systemic inflammatory response syndrome (SIRS), hematocrit, platelets, prothrombin time, albumin, aspartate aminotransferase (AST), glucose, serum creatinine, blood urea nitrogen (BUN), total cholesterol, high-density lipoprotein cholesterol (HDL), low-density lipoprotein cholesterol (LDL), triglyceride, serum calcium, C-reactive protein (CRP), and pleural effusion were significantly associated with the development of SAP, as inferred by univariate analysis.

**Table 2:** Comparison of clinical and laboratory findings between patients, with and without SAP in the training sample (487 patients).

Variable	No-SAP (n = 438)	SAP (n = 49)	P-value
Age, years (IQR)	46 (37-60)	51 (38-66)	0.115
Male sex, N (%)	270 (61.6)	31 (63.3)	0.825
Duration of symptoms (days, IQR)	1.8 ± 0.8	1.9 ± 0.8	0.799
BMI, kg/m^2^ (IQR)	23.4 (20.9-26.3)	24.4 (22.1-26.6)	0.083
SIRS, N (%)	157 (35.8)	34 (69.4)	<0.001
Biliary etiology, N (%)	190 (43.4)	17 (34.7)	0.243
Laboratory findings			
Hematocrit (l/l)	0.42 (0.38-0.45)	0.44 (0.41-0.49)	0.007
Platelets (10^9^/L)	202 (167-234)	184 (135-208)	0.005
Prothrombin time, s (IQR)	13.8 (13.1-14.6)	14.6 (13.6-15.3)	0.004
Albumin, g/L (IQR)	37.1 (33.3-39.3)	30.4 (27.5-33.9)	<0.001
Total bilirubin, mmol/L (IQR)	20 (14-31)	20 (15-28)	0.631
ALT, U/L (IQR)	43 (18-121)	48 (24-77)	0.868
AST, U/L (IQR)	36 (21-88)	60 (41-85)	0.005
Glucose, mmol/L (IQR)	7.7 (6.2-10.0)	10.2 (8.2-14.4)	<0.001
Serum creatinine, μmol/L (IQR)	63 (54-76)	81 (59-154)	<0.001
BUN, mmol/L (IQR)	4.6 (3.6-5.9)	7.3 (5.1-11.4)	<0.001
Total cholesterol, N			0.001
<160 mmol/L	203 (95.75)	9 (9.45)	
160-240 mmol/L	131 (83.97)	25 (16.03)	
>240 mmol/L	104 (87.39)	15 (12.61)	
HDL, mmol/L (IQR)	1.0 (0.8-1.3)	0.6 (0.4-1.0)	<0.001
LDL, mmol/L (IQR)	2.6 (2.0-3.3)	1.6 (1.3-2.4)	<0.001
Triglyceride (mg/dL), mmol/L (IQR)	1.3 (0.8-3.3)	2.4 (1.3-7.2)	<0.001
Serum calcium, mmol/L (IQR)	2.2 (2.1-2.3)	1.9 (1.6-2.1)	<0.001
C-reactive protein, mg/L (IQR)	30.5 (10.4-87.8)	80.0 (28.4-90.0)	0.003
Pleural effusion, N (%)	59 (13.47)	30 (61.22)	<0.001

Data were mean ± standard deviation, or numbers and percentages, or median (25th–75th percentile), as appropriate. N, number; IQR, interquartile range; BMI, body mass index; SIRS, systemic inflammatory response syndrome; ALT, alanine aminotransferase; AST, aspartate aminotransferase; HDL, high-density lipoprotein cholesterol; LDL, low-density lipoprotein cholesterol.

### Models Development, Calibration, Tenfold Cross-Validation on the Training Sample

**Figure 1:**
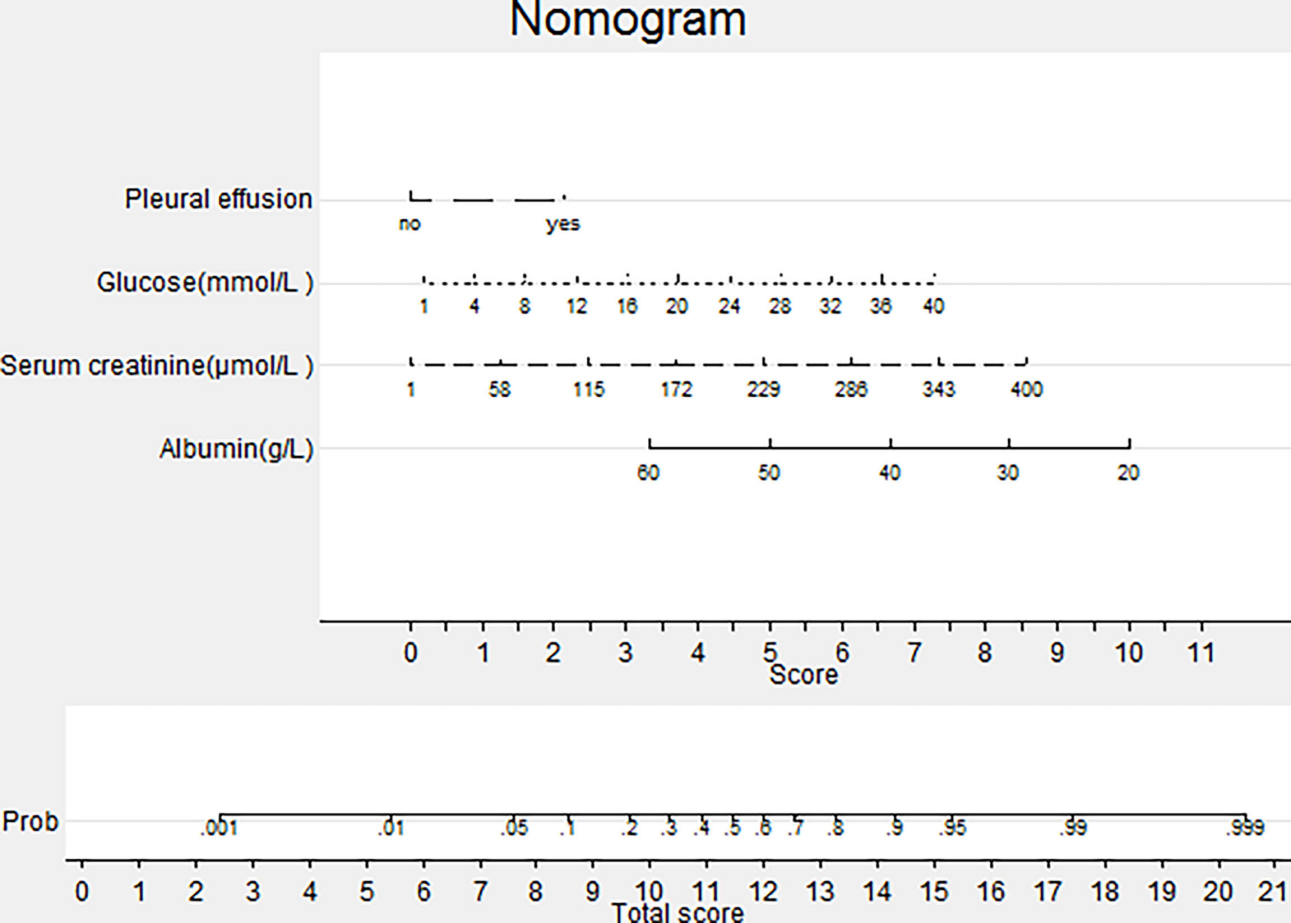
Nomogram predicting the probability of SAP. To obtain the nomogram-predicted probability, patient values on each axis were located and a vertical line was drawn to the point axis to determine how many points were attributed for each variable value. Points for all variables were summed and accessed on the point line to find SAP probability.

Variables significantly linked to the development of SAP in the univariate analysis were assessed by stepwise LR analysis. LR identified the following four independent variables as predictive of SAP: albumin (OR 0.88, 95%CI 0.81-0.95, P=0.002), serum creatinine (OR 1.02, 95%CI 1.01-1.03, P=0.002), glucose (OR 1.15, 95%CI 1.07-1.24, P<0.001), and pleural effusion (OR 5.11, 95%CI 2.38-10.94, P<0.001). The LR model was developed to predict SAP as the following function: -1.10-0.13×albumin (g/L) + 0.016× serum creatinine (μmol/L) +0.14 × glucose(mmol/L) + 1.63 × pleural effusion (0/1)(No/Yes). The coefficients of this formula were utilized to build a nomogram for the prediction of SAP **(** **Figure 1****)**. The Hosmer-Lemeshow goodness-of-fit test was significant (P=0.87), suggesting that our prediction model fit the actual data well.

**Figure 2:**
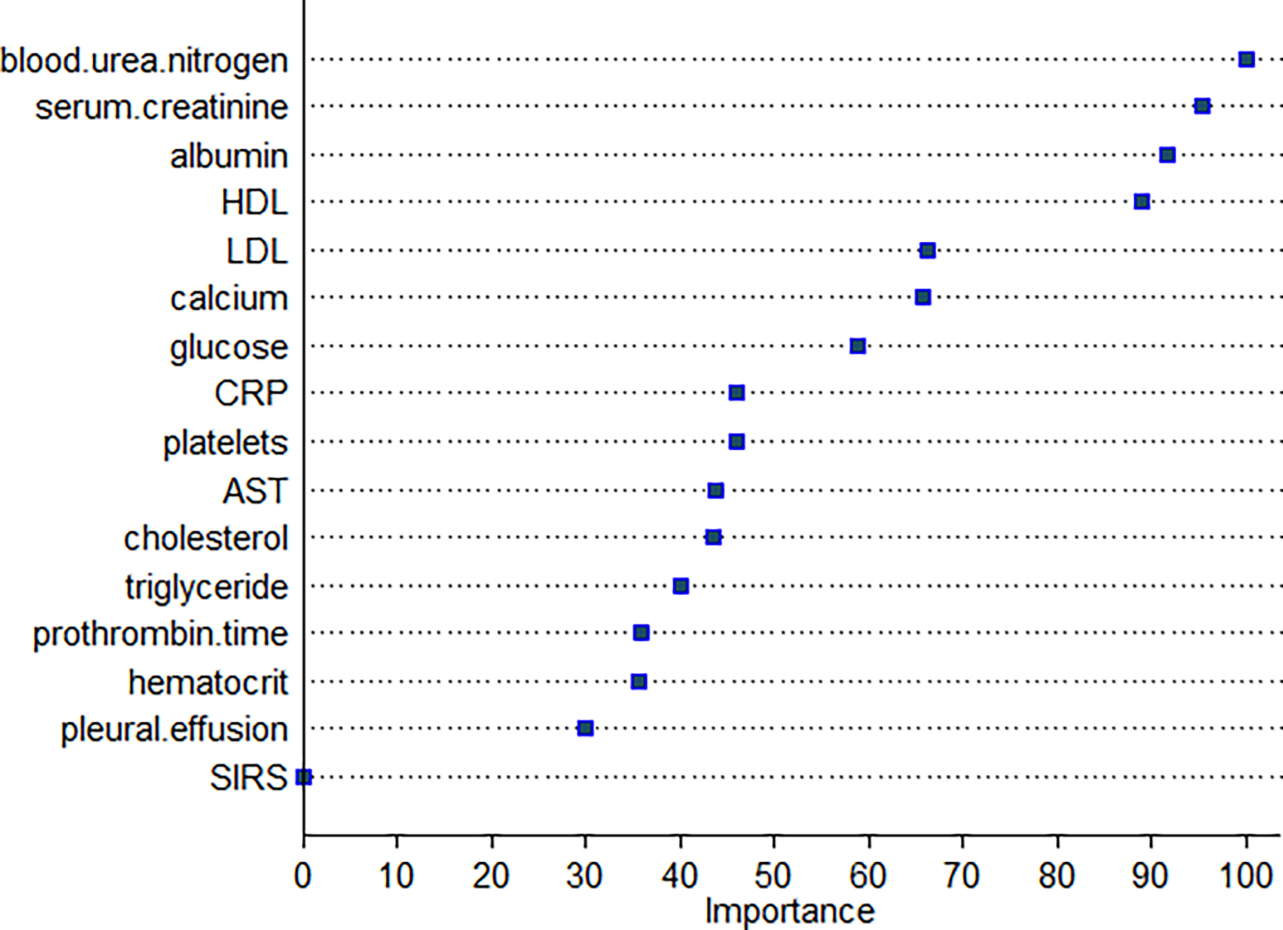
Variable importance plot of the RF for SAP.

**Figure 3:**
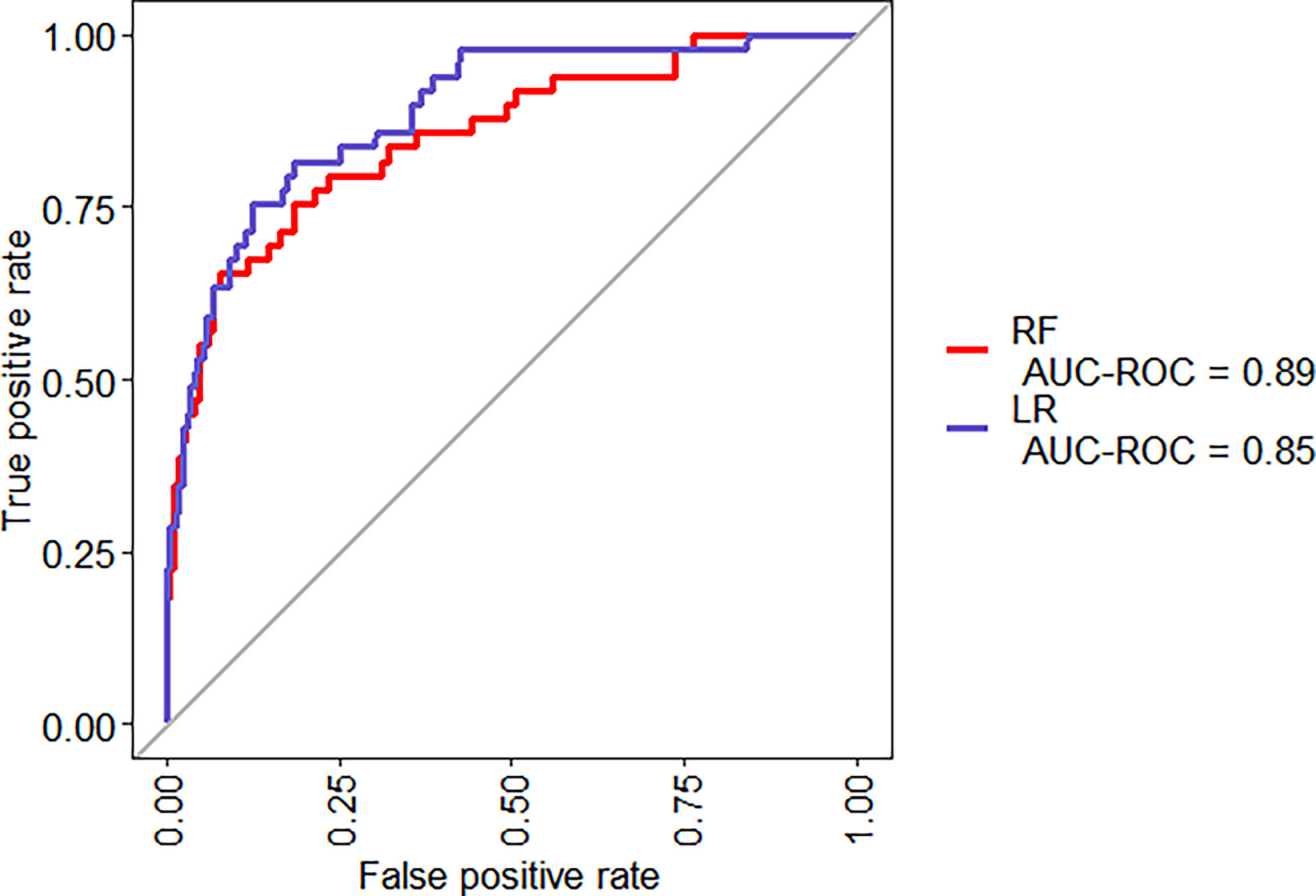
ROC curves for the RF and LR models, for a tenfold cross-validation on the training set.

**Figure 4:**
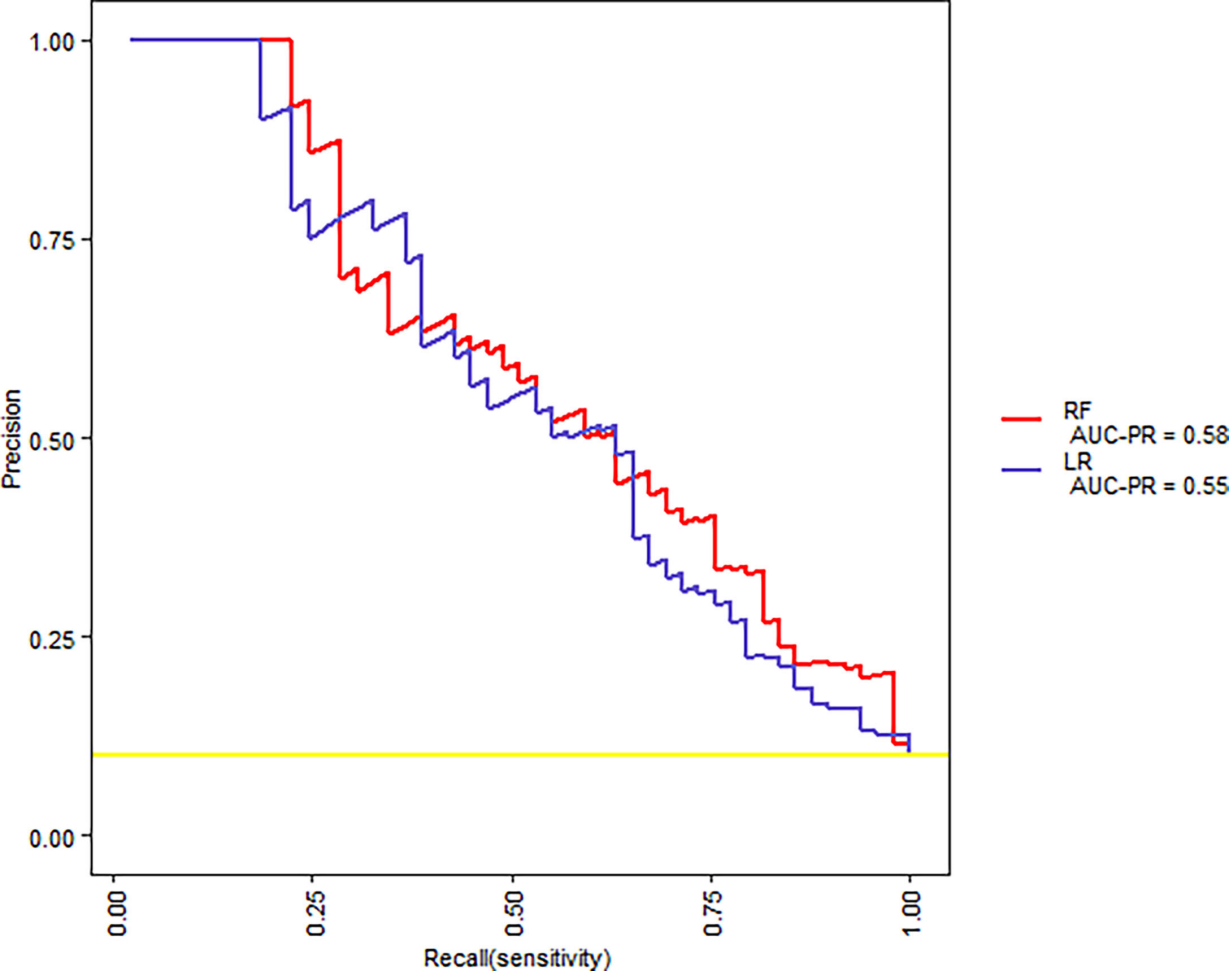
The precision-recall curves for RF and LR models for tenfold cross-validation on the training set.

**Figure 5:**
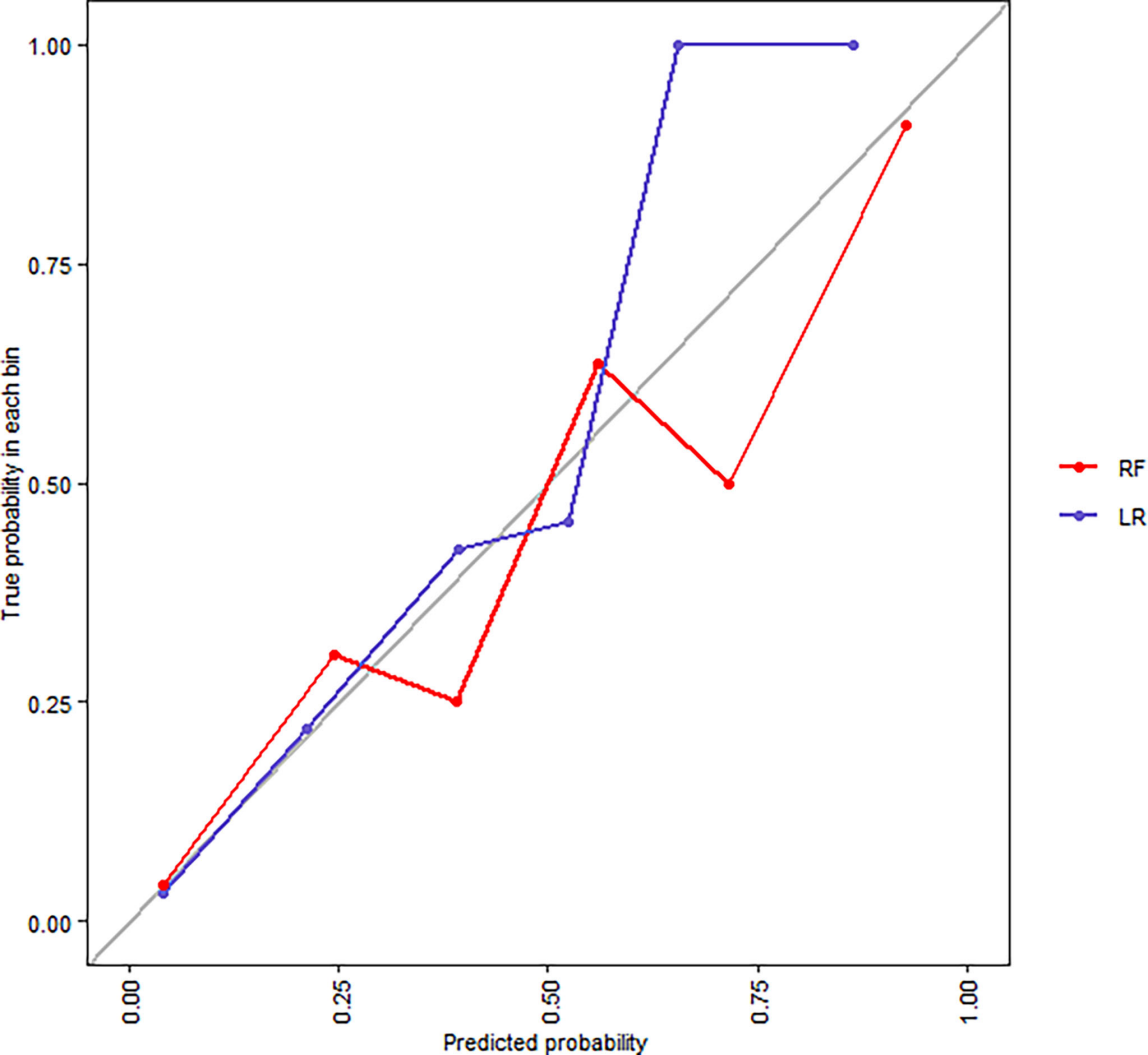
Calibration plots for RF and LR models for tenfold cross-validation on the training set.

The same 16 variables (SIRS, hematocrit, platelets, prothrombin time, albumin, AST, glucose, serum creatinine, BUN, cholesterol, HDL, LDL, triglyceride, serum calcium, C-reactive protein, and pleural effusion) were used for the RF model. As shown in **Figure 2**, based on variable important analysis of the RF model, serum creatinine, albumin, blood urea nitrogen, HDL, LDL, calcium, and glucose were the most important 7 predictors of SAP. **Figure 3** depicts the results of tenfold cross-validation. It indicated that the RF model achieved a higher mean AUC (AUC=0.89[95% CI, 0.83-0.95]) than that of the LR model (mean AUC =0.85[95% CI, 0.78-0.92]) (p=0.026). The area under the precision recall curve of the RF model (0.58) was also higher than that of the LR model (0.55) **(** **Figure 4****)**. The calibration plots indicate adequate predicted probabilities against observed proportions of SAP for both RF and LR models **(** **Figure 5****)**.

### Validation and Comparison of Prediction Models on the Test Samples

**Figure 6:**
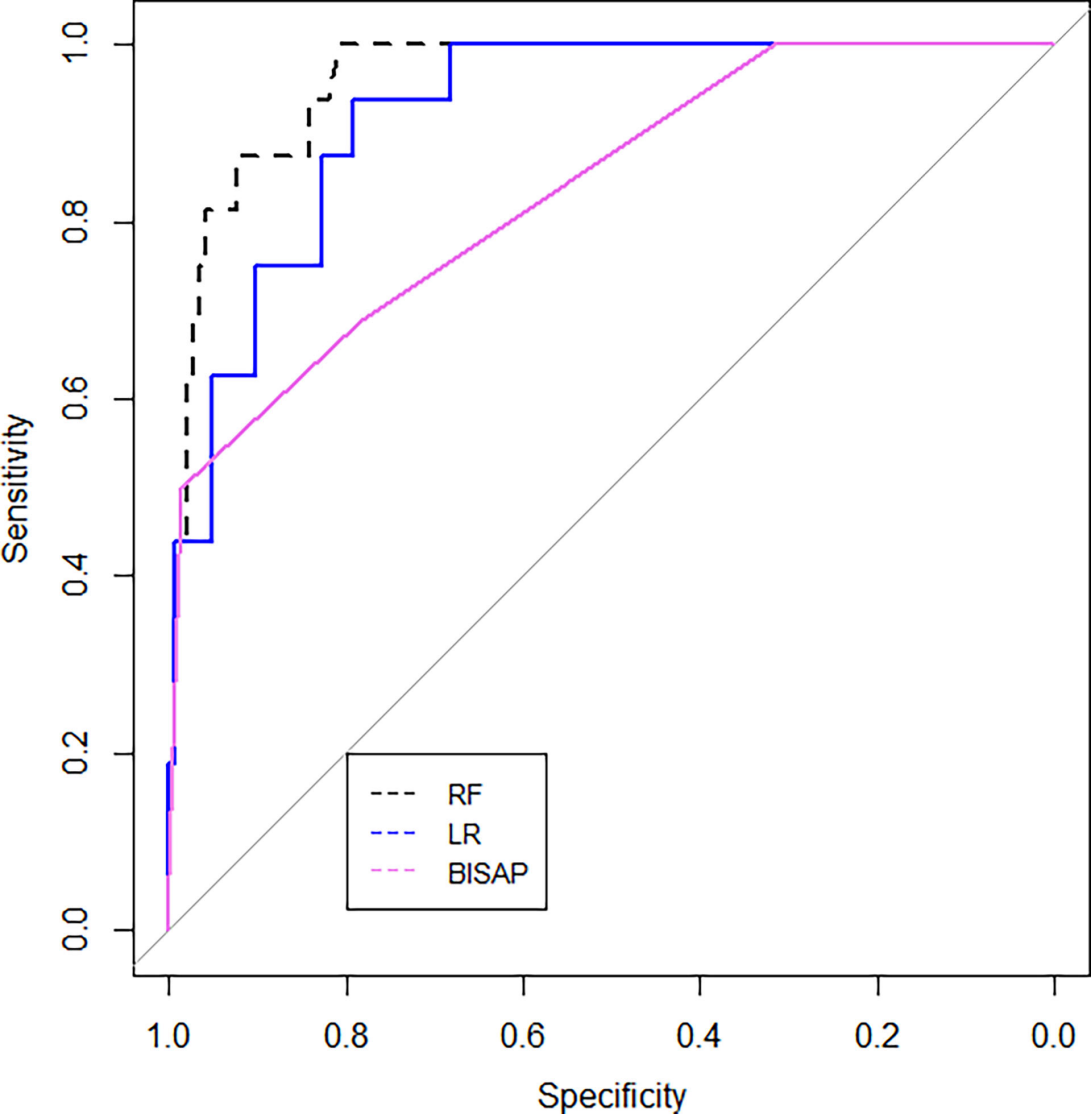
ROC curves for the RF and LR models and BISAP scores, applied on the test set.

**Figure 7:**
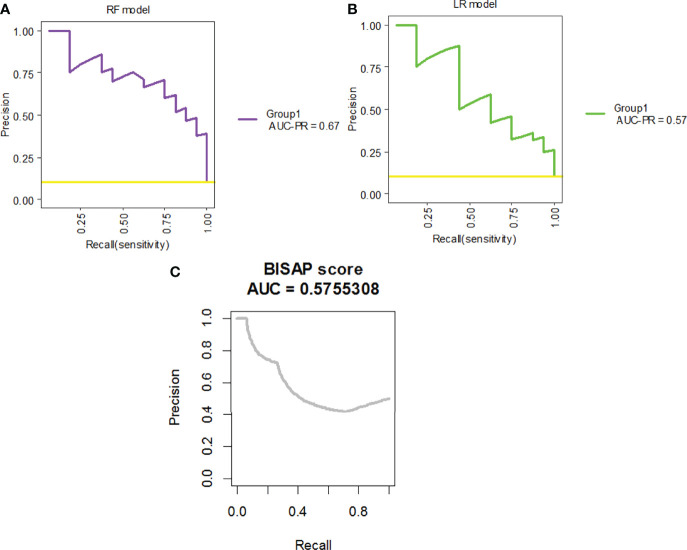
The precision-recall curves for the **(A)** RF model, **(B)** LR model, and **(C)** BISAP score applied on the test set.

The ROC curves for the RF model, the LR model, and the BISAP score for the prediction of SAP are shown in **Figure 6**. The RF model achieved the highest AUC (AUC=0.96[95% CI, 0.93-0.99]), followed by the LR model (AUC =0.92[95% CI, 0.87-0.97]) and the BISAP score (AUC=0.84[95% CI, 0.73-0.93]) (P=0.03). The area under precision recall curve of the RF model (0.67) was higher than that of both the LR model (0.57) and the BISAP score (0.576) **(** **Figure 7****)**.

The RF model achieved a sensitivity of 93.8%, specificity of 82.8%, and a diagnostic accuracy of 83.9%. As a comparison, the LR model achieved a similar sensitivity of 93.8%, a lower specificity of 79.3%, and 80.8% diagnostic accuracy. Both diagnostic performance of the RF and LR models was better than that of the BISAP score (**Table 3**).

**Table 3:** Diagnostic values of various models of SAP.

Variable	Cut-off value	Sensitivity	Specificity	LR+	LR-	Accuracy
RF model	0.13	93.8%	82.8%	5.44	0.08	83.9%
LR model	0.08	93.8%	79.3%	4.53	0.08	80.8%
BISAP score	2	68.8%	78.6%	3.22	0.40	77.64%

LR+, Positive likelihood ratio; LR-, negative likelihood ratio.

### Explanation: Individualized Prediction on The Test Sample

**Figure 8:**
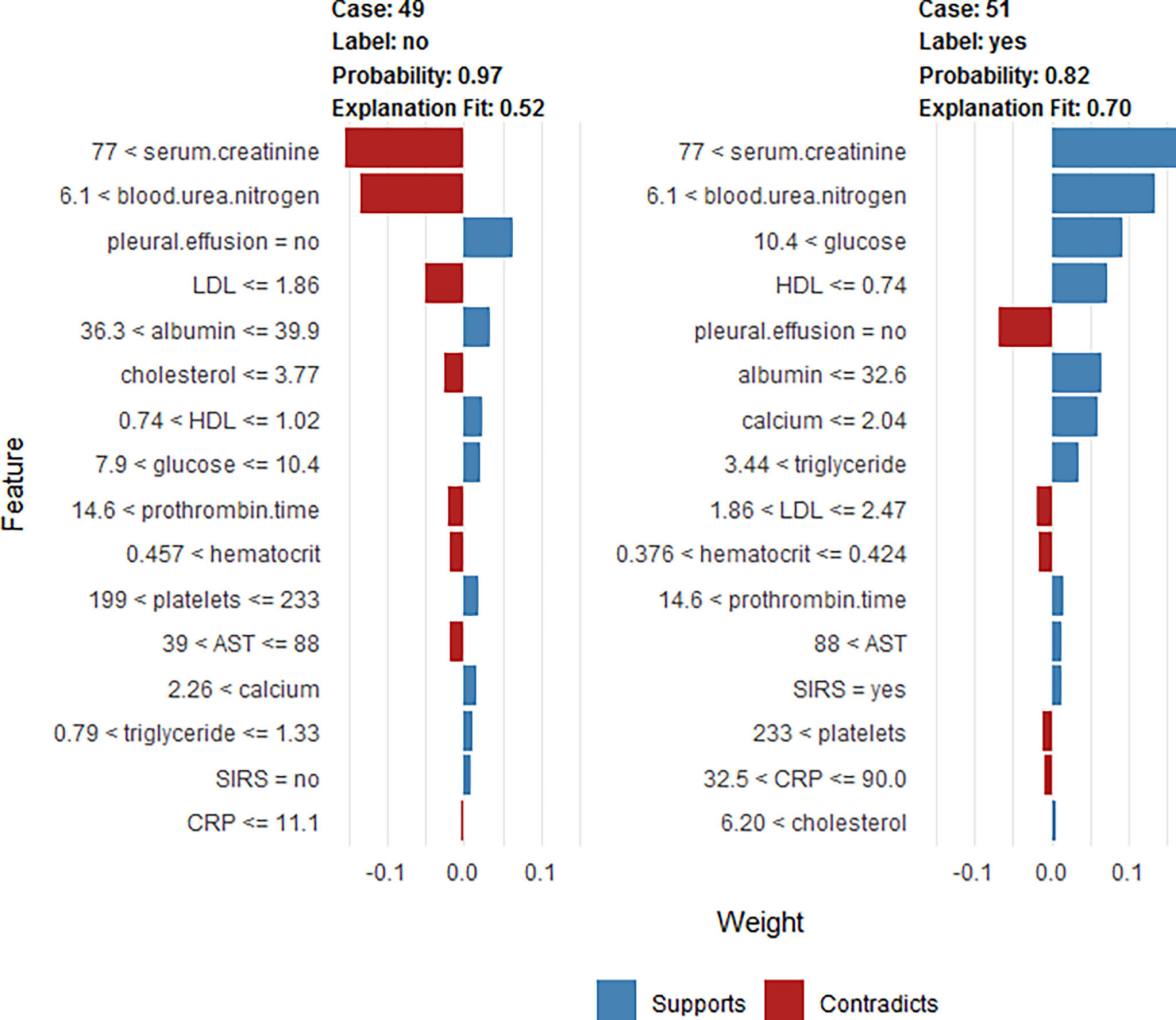
LIME plot for the individualized likelihood of two typical predictions. This shows the main contributing features behind the model prediction. The length of the color bar represents the amount of contribution. The first case (case 49) is a non-SAP patient who was correctly classified, with a prediction probability of 0.97 as non-SAP based on the RF model. The first case (case 49) had a creatinine value of 86 μmol/L, BUN=7.1 mmol/L, no pleural effusion, LDL=1.82 mmol/L, albumin=36.5 mg/dl, total cholesterol=3.24 mmol/L, HDL=0.79 mmol/L, glucose=8.4 mmol/L, prothrombin time=15.2 s, hematocrit=0.465, platelets=206×10^9/L, AST=76 U/L, calcium=2.43 mmol/L, triglyceride=0.96 mmol/L, no SIRS, and CRP=5 mg/L. The second case (case 51) is an SAP patient who was correctly classified, with a prediction probability of 0.82 (SAP based on RF model). The second case (case 51) had a creatinine value of 260 μmol/L, BUN=16.6 mmol/L, glucose =23.2 mmol/L, HDL=0.47 mmol/L, no pleural effusion, albumin =26.5 mg/dl, calcium=0.83 mmol/L, triglyceride=25.6 mmol/L, LDL=1.87 mmol/L, hematocrit=0.39, prothrombin time=15.7 s, AST=155 U/L, SIRS, platelets=243×10^9/L, CRP =76.1 mg/L, and total cholesterol=10.54 mmol/L.

To clarify the model prediction for individual patients, the LIME plot was generated. It shows two typical predictions made by the RF model, in which one was for non-SAP and the other was for SAP patients (**Figure 8**). The bar charts represent the influence that individual covariates have on the overall prediction (Chan et al., 2022). The length of the bar indicates the magnitude (absolute value), while the color indicates the sign (red for negative, blue for positive) of the estimated coefficient (Biecek and Burzykowski, 2021). In other words, the length of the bar for each feature indicates the importance (weight) of that feature in making the prediction. A longer bar, therefore, indicates a feature that contributes more toward or against the prediction (Lin et al., 2021a).

As shown in **Figure 8**, the first case (case 49) is a non-SAP patient, who was correctly classified based on the RF model, with a predicted probability of 0.97 as non-SAP. The second case (case 51) is an SAP patient, who was correctly classified based on the RF model, with a predicted probability of 0.82 as SAP. The levels of creatinine, BUN, glucose, triglyceride, and total cholesterol were positively correlated with the development of SAP. Patients with SAP had lower levels of HDL, albumin, and calcium than that of non-SAP people.

## Discussion

Albumin is one of the most important proteins in plasma and plays a role in maintaining osmotic pressure, antioxidants, and scavenging free radicals (Viasus et al., 2013). Albumin has also long been considered a negative acute-phase protein, with reduced production in inflammation, paving the way for inflammatory cytokines (Charlie-Silva et al., 2019). Serum albumin levels undoubtedly decrease in inflammatory states, which may result in shorter half-life and a larger interstitial pool (Barle et al., 2006) as well as capillary leak (Soeters et al., 2019), during the inflammatory process. Excessive oxidative stress is associated with damage to acinar cells which has been observed in cerulein-induced mouse models of AP (Shen et al., 2018). In addition, clinical evidence suggests that oxidative stress is common in the early phase of AP (Hackert and Werner, 2011). Therefore, it was suggested that decreased albumin may reduce the ability to counterwork oxidative stress-induced acinar damage by binding reactive oxygen species in AP (Xu et al., 2020; Belinskaia et al., 2021). Xu et al. has reported that albumin is an independent predictor for SAP and in-hospital mortality in AP patients (Xu et al., 2020). Our previous study also indicated that hypoalbuminemia within 24 h of admission is independently associated with the development of persistent organ failure and mortality in AP (Hong et al., 2017a). Ocskay et al. (Ocskay et al., 2021) reported that the incidence of hypoalbuminemia was 35.7% during hospitalization and it was dose-dependent, associated with severity and mortality in AP. In our current study, the LR analysis indicated that albumin (p<0.001) is an independent predictor of SAP **(** **Figure 1****)**. Based on the RF model, albumin is also an important predictor of SAP, based on variable importance analysis **(** **Figure 2****)**. These results are consistent with previous reports.

Creatinine is primarily generated by muscle mass and dietary intake. It is eliminated from the glomerular filtration membrane (Stevens et al., 2006; Earley et al., 2012) and serves as the most widely used functional biomarker of the kidney, which can reflect renal injury in AP (Earley et al., 2012). Apart from renal injury, it also has been reported that the level of serum creatinine is associated with pancreatic necrosis (Muddana et al., 2009; Papachristou et al., 2010; Lipinski et al., 2013). The possible explanation is that necrotic cells release a large number of toxic substances and pro-inflammatory factors to cause renal injury, manifesting the elevation of serum creatinine. Therefore, the rise of creatinine may be attributed to the renal injury and pancreatic necrosis along with SAP. Wilkman et al. reported that increased creatinine levels are independently associated with 90-day mortality in AP patients (Wilkman et al., 2013). Wan et al. suggested that serum creatinine levels within 24 h of admission are effective for predicting persistent organ failure in AP (Wan et al., 2019). In addition, several scoring systems, which take creatinine as an index, are widely used in the clinical settings, including the Acute Physiology and Chronic Health Evaluation (APACHE) II, Sequential Organ Failure Assessment (SOFA) score for predicting the severity of pancreatitis, and modified Marshall scoring system for assessing organ dysfunction occurrence in SAP (Mederos et al., 2021). Our study indicated serum creatinine could be a useful predictor in both the RF and the LR model for predicting SAP **(** **Figures 1**, **2****)**.

The lipoprotein profile, especially HDL, is markedly decreased in inflammation and the accompanying acute-phase (Jahangiri, 2010). The mechanisms causing low serum HDL and LDL levels in the acute phase of AP remain largely unknown (Hong et al., 2017b; Hong et al., 2018). Jahangiri et al. (Jahangiri, 2010) suggested that it was related to a decreased rate of lipoprotein synthesis in the liver, general catabolism, and activation of the inflammatory system in the acute phase of the disease. Another explanation for the low serum HDL levels is that it may be due to increased expression of the Toll-like receptors (TLRs), especially TLR-4 expression (Zhang et al., 2010). It was reported that stimulated TLR-4 expression suppresses HDL levels (Liao et al., 1999). Khan et al. found that serum lipid concentrations such as HDL cholesterol and LDL cholesterol were associated with patients of SAP in all etiologies (Khan et al., 2013). However, Bugdaci et al. (2011) found a significant association between decreased HDL level and severity of the disease only in alcoholic and hypotriglyceridemic pancreatitis. In hypertriglyceridemic status, it is demonstrated that free fatty acids (FFAs) damage acinar cells and cause pancreatitis attack due to premature activation of trypsinogen, by creating an acidic environment (Okura et al., 2004; Guo et al., 2019). It has been reported that HDL takes part in FFA clearance (Asztalos et al., 2007) so that decreased HDL in hypertriglyceridemic AP cases may lead to an increase in FFA, and further damage acinar cells. Therefore, it has been suggested that an increase in HDL may be helpful for recovery from the disease by contributing to antioxidants (Bugdaci et al., 2011) and anti-inflammatory effect (Murphy and Woollard, 2010). On the other hand, in comparison, few studies are available about the pathophysiological mechanism of decreased LDL in SAP. Our study indicated both HDL and LDL were useful predictors for SAP (**Figure 2**).

Pleural effusion occurs in 3–50% patients with AP, based on a previous study (Basran et al., 1987; Kumar et al., 2019; Peng et al., 2020). The effusion can be asymptomatic and often hemorrhagic, usually resolving as pancreatitis subsides (Basran et al., 1987). Several mechanisms of pleural effusion in pancreatitis have been proposed, such as the trans-diaphragmatic lymphatic blockage, the pancreatic pleural fistula caused by the rupture of the pancreatic duct, and the fluid exudation from the sub-pleural diaphragmatic vessels into the pleural cavity (Kumar et al., 2019). Pleural effusion is reported to be associated with a severe course for initial risk assessment severity in AP and a sign of SAP (Heller et al., 1997; Tenner et al., 2013; Lankisch et al., 2015). Yan et al. reported that pleural effusion volume quantified on chest CT was positively associated with the duration of hospitalization (Yan et al., 2021). As a prognostic factor, pleural effusion has been incorporated in SAP severity predictive systems such as the Bedside Index for Severity in Acute Pancreatitis (BISAP) score (Gao et al., 2015), the Panc 3 score (Brown et al., 2007), and the Extra Pancreatic Inflammation on CT (EPIC) score (De Waele et al., 2007). Following the above outcomes, the present study suggested that pleural effusion (OR 5.11, 95%CI 2.38-10.94) was an independent risk factor for SAP (**Figure 1**).

The mechanism of BUN elevation in AP is thought to be based on the loss of intravascular volume, caused by interstitial extravasations owing to the systemic inflammatory response syndrome and an AP promoted direct renal injury mechanism. It has been reported that BUN, as a single predictor, had moderate accuracy in predicting persistent organ failure in AP (Mounzer et al., 2012). Koutroumpakis et al. reported that the rise in BUN at 24 h was the most accurate in predicting persistent organ failure and pancreatic necrosis (Koutroumpakis et al., 2015). Li et al. suggested that BUN was an independent risk factor to predict in-hospital mortality (Li et al., 2020). Valverde-Lopez et al. indicated that BUN was the best predictor of SAP after 48 h (Valverde-Lopez et al., 2017). BUN is also included in many scoring systems for AP, such as BISAP, JSS, and Glasgow score (Mounzer et al., 2012). Consistent with these reports, our study shows that BUN is the most important predictor of the RF model based on variable important analysis (**Figure 2**).

Decreased levels of serum calcium are commonly seen in critical illness, and hypocalcemia is significantly more frequent in patients with SAP (Peng et al., 2017). The mechanisms of hypocalcemia in SAP may be multi-factorial, such as abnormalities of parathyroid hormone secretion and action as well as vitamin D deficiency, binding of calcium in areas of fat necrosis, likely to contribute to the medication side effects (Weir et al., 1975; Steele et al., 2013). Serum calcium levels are closely related to the severity of the disease and its complications in AP. It has been incorporated in several clinical scoring systems as such as Pancreatitis Outcome Prediction (POP) Score, and Simple Prognostic Score (Harrison et al., 2007; Gonzálvez-Gasch et al., 2009). Mentula et al. suggested that serum calcium was the best single marker in predicting organ failure in AP after 24 h of symptom onset (Mentula et al., 2005). He et al. indicated that serum calcium was one of the independent predictors of the severity of AP in elderly patients (He et al., 2021). Serum calcium was also considered a significant factor in predicting early death in SAP (Shinzeki et al., 2008). As expected, our study indicated that calcium could be a useful predictor of SAP in the RF model (**Figure 2**).

Clinical evidence shows hyperglycemia is the common early feature of AP and abnormal glucose metabolism is present in almost 40% of AP patients (Banks et al., 2013; Chen et al., 2021). According to the traditional view, the mechanism is that the damage of organisms caused by AP activate the neuroendocrine system and lead to the secretion of many stress hormones (Binker and Cosen-Binker, 2014; Sun et al., 2019; Lu et al., 2021). Meanwhile, it is also related to the damage of the endocrine pancreas caused by SAP attacks. The association between hyperglycemia and adverse clinical outcomes in critically ill patients has been demonstrated in several observational studies, which suggest that high levels of glucose during the progression of AP can promote the release of inflammatory cytokines. These, in turn, influence disease progression (Sun et al., 2019; Chen et al., 2021). Sun et al. has suggested that the level of glucose in serum is positively correlated with the APACHE II scores, TNF-α, and CRP in AP (Sun et al., 2019). However, transient stress hyperglycemia in critically ill patients is considered harmless in some studies, indicating that the body has normal immune regulation ability (Lu et al., 2021), the subsequent derangement of glucose homeostasis could cause damage to the body (Pendharkar et al., 2016). Blood glucose-related indicators are associated with in-hospital mortality in critically ill patients with AP (Lu et al., 2021). Our LR model also shows that glucose is a useful predictor of SAP (**Figure 1**).

Machine learning has been extensively used for the prediction of severity or complication of AP (Zhou et al., 2022). Thapa et al. has reported that an XGBoost model could predict which patients would require treatment for SAP (Thapa et al., 2022). Early prediction of SAP using machine learning has also been attempted (Thapa et al., 2022). Jin et al. reported that the multilayer perception-artificial neural network (MPL-ANN) model based on routine blood and serum biochemical indexes could reliably predict disease severity in patients with AP (Jin et al., 2021). Choi et al. combined clinical (i.e., APACHE-II and BISAP scores) and radiologic (i.e., Balthazar grade and EPIC score) scoring systems by classification tree analysis for predicting SAP (Choi et al., 2018). Xu et al. reported that adaptive boosting algorithm (AdaBoost) could predict development of multiple organ failure, complicated by moderately severe or severe AP (Xu et al., 2021). However, the above models were limited due to lack of individualized prediction on the test sample. Implementation on such data remains challenging because of the low interpretability of results of machine learning (Yu et al., 2021). Our study indicated that, compared to the LR model and BISAP score, RF exhibited the highest discriminatory performance for the prediction of SAP on both training and test samples (**Figures 3**, **4**, **6**, **7**). Using the RF model, we could illustrate key features and establish a prediction model, with high accuracy in patients with SAP. The LIME plot could provide a visual illustration of the individualized interpretation of the importance of different features, which might help clinical doctors to understand results of the RF model (**Figure 8**). The LR model (nomogram) achieved a sensitivity of 93.8%, acceptable specificity of 79.3%, and diagnostic accuracy of 80.8% (**Table 3**). Though the diagnostic performance of the LR model (nomogram) is inferior to the RF model, it is simple and intuitive to calculate the prediction probability of a result, which makes it valuable in predicting SAP (**Figure 1**).

To the best of our knowledge, this is the first study to develop an interpretable RF model for SAP prediction. The strength of this study is a large sample size, which enables a strong statistical power. Both patients in ICU and the general ward were enrolled in this study, thus reducing selection bias. However, our study has some limitations, even if it has been internally validated by tenfold cross-validation technique and test set, testing the performance of our RF model in an external/other independent data set is necessary. In addition, even if effective, RF models are sophisticated and difficult to understand, and thus, comparable to a ‘black box’. We have, therefore, demonstrated that by utilizing Lime plots, the results could be more easily interpreted (Al’Aref et al., 2020). At last, we did not evaluate the RF model and single predictors for other clinical outcomes such as patient survival and organ failure occurrence, intensive care unit (ICU) admission, and SAP recurrent rate. It would be interesting to carry out a large-sample prospective study to determine whether our model and other variables such as serum creatinine, albumin, BUN, HDL, LDL, calcium, and glucose play a significant role in predicting these clinical outcomes.

In conclusion, an interpretable RF model exhibited the highest discriminatory performance to predict SAP. Interpretation with LIME plots could be useful for individualized prediction in the clinical setting. A nomogram consisting of albumin, serum creatinine, glucose, and pleural effusion is also useful for the prediction of SAP.

## Data Availability Statement

The raw data supporting the conclusions of this article will be made available by the authors, without undue reservation.

## Ethics Statement

The studies involving human participants were reviewed and approved by Ethics Committee of the First Affiliated Hospital of Wenzhou Medical University. Written informed consent for participation was not required for this study in accordance with the national legislation and the institutional requirements.

## Author Contributions

WH conceived the study and carried out the majority of the work. WH participated in data collection and conducted data analysis. WH, YL, XZ, SJ, JP, QL, and SY drafted the manuscript. ZB, MZ, and HG helped to finalize the manuscript. All the authors read and approved the manuscript.

## Funding

This work was supported by Zhejiang Medical and Health Science and Technology Plan Project (Number: 2022KY886), Wenzhou Science and Technology Bureau (Number: Y2020010).

## Conflict of Interest

The authors declare that the research was conducted in the absence of any commercial or financial relationships that could be construed as a potential conflict of interest.

## Publisher’s Note

All claims expressed in this article are solely those of the authors and do not necessarily represent those of their affiliated organizations, or those of the publisher, the editors and the reviewers. Any product that may be evaluated in this article, or claim that may be made by its manufacturer, is not guaranteed or endorsed by the publisher.

## Supplementary Material

Supplementary Figure 1 Data flow diagram of this study.
