# Supplementary figures and images for: Usefulness of Random Forest Algorithm in Predicting Severe Acute Pancreatitis
Source: Front Cell Infect Microbiol. 2022 Jun 10;12:893294. doi: 10.3389/fcimb.2022.893294 (PMC9226542; doi:10.3389/fcimb.2022.893294)

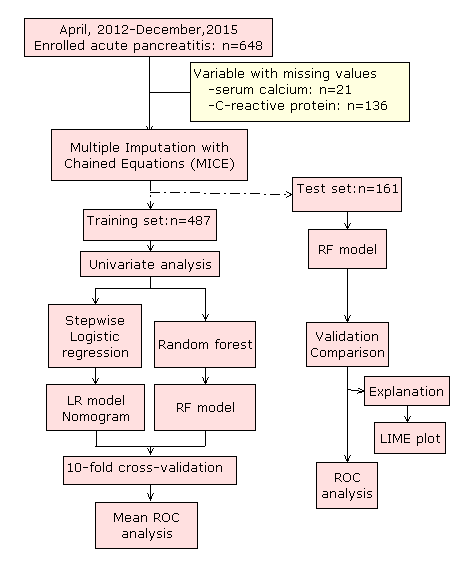

Supplement: Supplementary Figure 1 — Data flow diagram of this study. [file Image_1.tif]
